# Moving Beyond the Gym: A Content Analysis of YouTube as an Information Resource for Physical Literacy

**DOI:** 10.3390/ijerph16183335

**Published:** 2019-09-10

**Authors:** Trevor Bopp, Joshua D. Vadeboncoeur, Michael Stellefson, Melissa Weinsz

**Affiliations:** 1 Department of Sport Management, University of Florida, Gainesville, FL 32611, USA; 2 Department of Health Education and Promotion, East Carolina University, Greenville, NC 27858, USA

**Keywords:** physical literacy, activity, social media, online resource, Internet, HONcode, YouTube

## Abstract

The Internet, and particularly YouTube, has been found to be and continues to develop as a resourceful educational space for health-related information. Understanding physical literacy as a lifelong health-related outcome and facilitator of an active lifestyle, we sought to assess the content, exposure, engagement, and information quality of uploaded physical literacy videos on YouTube. Two researchers collected 300 YouTube videos on physical literacy and independently coded each video’s: title, media source of upload, content topics related to physical literacy, content delivery style, and adherence to adapted Health on the Net Foundation Code of Conduct (HONcode) principles of information quality. Physical literacy videos that focused on physical activity and behaviors were the strongest predictor of high quality ratings, followed closely by videos covering affective domains (motivation, confidence, and self-esteem) of physical literacy. The content delivery method was also important, with videos utilizing presentations and testimonials containing high quality information about physical activity. Thus, providers of physical literacy and health-related online video content should be aware of and adhere to the expected quality standards. As health information expectations and ethical standards increase, the Internet, and specifically YouTube, has the potential to enhance video resources, virtual networking opportunities, as well as the sharing, dissemination, accumulation, and enrichment of physical literacy information for all.

## 1. Introduction

Regular physical activity is a critical component of healthy living, particularly as it pertains to weight management, preventing chronic disease, and promoting psychological well-being [1,2]. It is especially important for youth to become physically active so as to mitigate health risk factors that can accumulate and negatively impact health outcomes over life course trajectories [3,4]. For example, obesity affects nearly 1 in 5 American children and adolescents [5]. Struggles with obesity and weight status present youth with challenges to performing basic movement skills [6], which subsequently impacts their self-confidence, and in turn, deters their participation in activities that are likely to develop fine motor skills necessary for physical activity [4]. Physical literacy is a foundational and enduring concept regarding one’s “motivation, confidence, physical competence, knowledge, and understanding to value and take responsibility for engagement in physical activities for life [7]”. Given its conceptualization as the basis for a life course trajectory of healthy living, physical literacy has evolved into a relevant lifelong learning outcome for all individuals, of all ages and ability levels.

According to Whitehead [8], the affective domain of physical literacy refers to the relationship among an individual’s confidence, motivation, and self-esteem in relation to their level(s) of physical activity. Additionally, physical literacy is contingent upon the cognitive accrual and maintenance of, “knowledge and understanding to value and take responsibility” for engaging in physical activity across the life course [7]. Knowing how to live an active lifestyle establishes the cognitive foundation for an individual to become aware of and attach value to participation in physical activity [9]. Thus, physical literacy should influence attitudes toward physical activity, wherein “attention to understanding the importance of physical activity, health and wellbeing throughout the life course [10] (p. 120)” becomes of prime importance.

At present, scholarly conversations continue as to how to best implement, endorse, and teach physical literacy in educational settings and sport/physical activity programs [11,12]. For instance, the Society of Health and Physical Educators (SHAPE) provides resources and professional development opportunities to help youth develop into physically literate individuals, yet still faces challenges when attempting to “describe and demonstrate what physical literacy informed practice is and looks like without being overly perspective and restrictive [11] (p. 270)”. A number of international organizations (e.g., Aspen, Canadian Sport for Life [CS4L], International Physical Literacy Association [IPLA]) also offer online platforms that provide informational resources, scholarly publications, strategies for promoting and implementing physical literacy curriculum and standards, and instructor training workshops on physical literacy. Despite the wealth of information made available online by these organizations physical educators, teachers, and those with a general interest in learning remain at a loss when searching for online physical literacy resources [13].

When considering physical literacy from a health perspective, both as an outcome and antecedent to an active and healthy lifestyle, it becomes a worthwhile endeavor to examine and understand how physical literacy information is shared and disseminated on the Internet, and in particular, among social media users. Social media has been found to be a popular and increasingly-accessible resource for health-related information [14]. YouTube is a public platform and people from wide ranging disciplines and contexts access it for information on broad range of topics and issues. As a continually developing online resource for health information, communication, and promotion, health education researchers have examined the use and effectiveness of specific popular social media sites such as YouTube [15,16,17,18], Pinterest [19,20], Instagram [21], and Facebook [22]. Given the popularity, reach, accessibility and unregulated governance of social media, it is critical that users seek and post/upload information from reputable, qualified and trusted health and medical sources. Therefore, the following section further details social media as a health-related information platform, with particular attention paid to YouTube.

### Social Media and YouTube as Information Resources

As the Internet and social media allow for asynchronous and synchronous transmission and dissemination of information, it is critical for proponents of physical literacy (e.g., teachers, parents, students, etc.) to be aware of how physical literacy is portrayed online, especially through popular social media which is a common learning forum for youth health and wellness [23]. Given that youth have been found to place a relatively high value on the accessibility of information made available on social media [24], extent research has demonstrated that youth are not only pivoting to social media platforms for health-based information at increasing rates [14], but in doing so, are reporting benefits such as higher levels of socialization, knowledge procurement, and even socio-psychological support [25,26]. Likewise, it is in this vein that many individuals of adult-age may hold an unawareness of the potential opportunities that social media embodies as an emerging medium for health promotion amongst youth, much of which can be attributed to the complex nature behind how social media operates to influence youth [24]. For example, given the breadth of social media platforms (e.g., Facebook, Instagram, Snapchat, Twitter, YouTube), interactive applications and features made available to engage with said platforms, and “who” and “what” is accessing and/or creating content, each operates in conjunction to offer a contextually dynamic space that diverges from what is considered to be more traditional forms of public health pedagogies [27,28].

Nevertheless, as posited by Dudley et al. [29], “developing health literacy in young people is an important construct that should be considered in the context of engagement with social media,” such that it can offer adolescents, “greater availability of information and with the potential for shared dialogue and positive normative beliefs that enhance healthy behaviors” (p. 157). Although in agreement with the aforementioned sentiments, Cale [30] cautioned that because health and physical education teachers and practitioners alike remain focused on instrumental outcomes rather than health benefits, the use of social media in physical education pedagogy may very well serve as yet another platform that replicates and reinforces the same static approaches already in practice. Furthermore, there is a lack of sourcing of health information from digital technologies on behalf of teachers and practitioners [31,32,33], thus prompting growing recommendations in the research literature for an intentional, “focus on adults’ critical pedagogical response both to health and health-related social media,” as means “to develop critically aware pedagogues who can support and develop critically aware youngsters” [30] (p. 216).

Moreover, as noted by Casey, Goodyear, and Armour [33], among the practitioners in their study, digital technologies were merely used to reinforce traditionally-held physical education pedagogical practices, the likes of which could be extrapolated to include the interrelationship(s) between social media technologies, health, and physical education [30]. It is no wonder that recent scholarship has placed an intentioned focus on the use of social media [34] and related emergent technologies [35] in the promotion of health-related and physical education information, as well as the bridging of physical education and digital technology through pedagogical (re)development [36,37]. For instance, and more narrowly, extant literature has deemed platforms such as Facebook, for example, to be an effective information resource in diverse contexts, such as through pedagogical design [38,39,40]) and more pointedly, for the purposes of sharing health information, both at the individual [41] and organizational [42] levels. As a result, understanding the dynamic functionality and interactivity of social media platforms, particularly concerning their influence on the health-related behavior(s) and knowledge of youth, becomes imperative. Thus, we are in alignment with Shaw, Mitchell, Welch, and Williamson’s [43] assertion that all future inquiry on social media as a health intervention and information resource for youth must consider “the newness of using social media as a health intervention, the importance of the use of rigorous methodological processes when using social media as a health intervention, and the need to develop further knowledge on adolescents’ use of social media (p. 8)”.

While the aforementioned discussion demonstrates the recent (and yet, ongoing) investigation of social media platforms as health-related, information resources, little attention has been directed towards evaluating physical literacy videos on YouTube. For instance, although YouTube has been investigated as a source of information for a number of health-related topics [15,16,17,18,44,45], as well as a teaching aid in physical education [46], physical literacy has been absent from this line of inquiry. YouTube (www.youtube.com), with 1.8 billion registered users [47], is the largest and most utilized video-based social media platform globally [48]. The platform allows users to view, upload and rank video content as well as connect and communicate will fellow users. It is the second most popular search engine behind Google [48] and is used by 73% of U.S. adults, 94% of U.S. 18- to 24-year-olds, and 85% of U.S. teens ages 13–17 [49,50]. YouTube has been identified as a valuable learning resource that, when properly integrated into school settings, assists in the daily delivery of curriculum. Its broad reach and engagement capacity makes it the ideal technology for serving as a supplemental learning channel that extends the classroom environment beyond the school walls and into natural physical activity settings [51]. Furthermore, YouTube is one social media platform notably utilized for the sharing of patient education materials and healthcare information [15,16,18]. Thus, the purpose of this study was to assess the content, exposure, engagement, and information quality of uploaded YouTube videos on physical literacy. To this end, we address the following research questions regarding physical literacy videos posted on YouTube:

1. Is the information quality of YouTube videos on physical literacy associated with viewer attitudes?
2. Does the quality of physical literacy information in the YouTube videos vary based on media source?
3. Do the content properties in the physical literacy YouTube videos influence the (a) quality of information and (b) viewer attitudes?
4. Does the content delivery style of the physical literacy YouTube videos influence the (a) quality of information and (b) viewer attitudes?

## 2. Materials and Methods

According to Petrescu [52], on average, 71% of organic desktop searches in Google result in web users clicking on results from the first page, whereas a click-through rate of just under 6% exists for page two and three results. Moreover, on the first page alone, 68% of web users will click on results within the first five listings, as opposed to results 6 through 10, which account for only 4% of all clicks. As such, to account for user behavior and ensure that a screening method devoid of arbitrary page and/or video count limits was employed, we utilized the “playlist” feature on YouTube. This feature allows an account user to curate a playlist of up to a maximum of 150 selected videos. While no methodology can provide truly objective data collection, we believed the utilization of YouTube playlists to be an appropriate standard from which to conduct this study. Thus, on September 11, 2018, two members of the research team independently conducted an initial video search, whereby the keyword “physical literacy” was input into the YouTube search engine.

For each video, we wanted to know which properties of physical literacy were addressed and in which context, athletic or educational; as well as the media source that uploaded the YouTube videos and for which target audience they were intended. To address these questions, the following details were recorded for each retrieved video: title, media source of upload, date of upload (measured in number of days since initial posting), duration (measured in minutes, seconds), content topics related to physical literacy (e.g., affective, cognitive, physical), the style of content delivery (e.g., animation, presentation, demonstration), and adherence to adapted Health On the Net Foundation (HONcode) principles of video quality (measured in frequency of principles followed).

As it concerned the media source of upload, we assigned each video to one of seven total categories: (a) educational institution (e.g., university, research institute, primary/secondary school); (b) government agency/organization (e.g., local sport council, parks and recreation department, government-sponsored); (c) news media outlet; (d) non-profit organization; (e) physical education-based/development program (e.g., for-profit organization offering physical education-based services); (f) professional organization (e.g., teachers association, professional development/learning); and (g) user-generated content (UGC).

We were also interested in the quality of physical literacy information in the YouTube videos. Efforts to best determine and measure the quality of information via ethical guidelines for internet-derived health information, such as self-regulatory codes of ethics, rating tools, third-party reviewers, and both accreditation and certification systems, have been numerous and diverse in their approaches [53,54]. Yet, one of the more popular and industry accepted tools, HONcode, was selected for this study. HONcode is a code of ethics for web site managers to follow as it concerns the presentation of objective and verifiable medical and health-related information. Extant research reveals that the HONcode has been well accepted by health-related web sites on account of its comprehensive set of principles that assess the ethics, quality, transparency, and trustworthiness of online-derived health information [55,56]. Additionally, due to the relative lack of assessment tools for the evaluation of online platforms such as YouTube [57], Stellefson et al. [18] demonstrated the utility of the HONcode as such a tool, using an adaptation of the instrument to measure information quality of Chronic Obstructive Pulmonary Disease (COPD) patient education videos posted to YouTube.

In accordance with each of the eight HONcode principles and subsequent adaptations [18], we used six adapted principles to evaluate the quality of selected YouTube videos. Although HONcode was developed as a code of ethics for web site content, we followed the similar efforts of Stellefson et al. in adjusting the principles in a manner that would render applicability to video content, which included thematic alignment with physical literacy and the collapsing of certain principles deemed to be unsuitable for this particular study. For each video, the coder indicated on a binary scale (0 = No; 1 = Yes) whether or not each of the six principles was followed, at which point a summated score was computed to measure total adherence to the adapted HONcode principles. Again, given the respective aims of this study, one of which is to assess the relative quality of information in videos shared on YouTube, we believe the merits of the HONcode principles and our subsequent adaptation to be best suited to carry out our data collection.

Additional metrics collected included total video view counts, viewer engagement (e.g., the number of “likes” and “dislikes”), and number of posted viewer comments. As per YouTube’s “Creator Academy” platform, a total of 18 example categories (e.g., education, entertainment, people & blogs, sports) are listed for content creators to organize and optimize the viewer reach of their videos. Thus, coders screened the listed category for each video (according to YouTube guidelines, only one category can be selected per video) and evaluated the relevance of the selected category per the video’s content topic.

As it concerns the categorical organization of both content topics and the delivery of said content, the authors collaborated to devise a definitive list for each set of categories. In drawing upon Edwards et al. [10] systematic review of the physical literacy construct, we developed a set of adapted content subthemes and core categories (noted in parentheses), which included: affective (confidence, motivation, self-esteem); cognitive (knowledge and understanding, value and responsibility); physical capabilities (FMS/capacity to move, competence); target audience(s) (youth, adults, none); behavioral characteristics (health behaviors, physical activity); psychological, social, and attitudinal (academic performance, enjoyment, support); contextual (structural or unstructured sport, physical education); and additionally, the subthemes of pathway, environment, holistic/ontological, and pedagogical. Several categories were first utilized when assessing the style of content delivery, however, after discussing the perceived intentions and outcomes of each delivery style category, we collapsed them into four primary classifications: animation (e.g., picture, script); presentation (i.e., academic, in classroom, conference); demonstration (i.e., visual observation, instructional); and testimonial (e.g., organizational, individual).

In order to satisfy the inclusion criteria of this study, video content had to be audible in the English language and pertinent to physical literacy content topic. Both coders were informed of the respective criteria for locating and henceforth evaluating each video. For each video that satisfied our inclusion criteria, both coders saved the video to a YouTube playlist. As previously indicated, a maximum of 150 videos can be saved on a single playlist, which allowed the coders to save a total of 300 videos. Of those selected, all videos with content that did not relate to the aims and scope of this study (*n* = 22), were not in English (*n* = 37), and duplicate videos (*n* = 91) were subsequently excluded. Whenever disagreement arose between both coders as to whether a particular saved video did not meet the inclusion criteria, resolution was met by either common consensus through discussion or by way of a third member of the research team. In all, a total of 150 unique videos were saved and utilized for data analysis.

### 2.1. Data Reliability

Interrater reliability of codes was established to confirm an appropriate level of consensus between the two coders. To determine interrater reliability via Cohen’s Kappa (ĸ), or coefficient of agreement, 40 videos were randomly selected and coded by two independent researchers. A subsample size of 40 was deemed appropriate for conducting reliability analyses [58] and the ĸ coefficient cutoff was set at the moderately acceptable value of 0.60 [59]. Using IBM SPSS software version 25 [60], coefficients of agreement were computed for coding the properties of physical literacy (ĸ = 0.82), target audiences (ĸ = 0.90), contexts (ĸ = 0.77), and delivery style (ĸ = 0.83). For a further breakdown of the reliability analyses see Table 1. Additionally, reliability of the adapted physical literacy HONCode principles was established using Lin’s concordance correlation coefficient [61] and was determined to be almost perfect, ρ_c_ = 0.90. This metric was selected as it can serve as a robust measure between two coders when observing continuous variables [18,62].

### 2.2. Data Analysis

IBM SPSS software version 25 [60] was utilized to run basic frequency and descriptive statistics to understand more about the affective, cognitive, behavioral, physical, psychological, social and attitudinal properties of physical literacy covered in the YouTube videos. Likewise, the target audiences, athletic/educational contexts, and media source of the YouTube videos were also examined via frequency and descriptive statistics. The quality of each video was determined by the adding together the number of adapted HONcode principles that each of the physical literacy YouTube videos met; adherence to 0–3 principles indicated low quality, while 4–6 principles suggested high quality [18]. Due to the non-normal distribution of data, the Mann-Whitney *U* test was conducted to determine associations between video quality of information and viewers’ attitudes, via “likes” (Research Question 1). Similarly, Fisher’s exact test was conducted to determine associations between media source of the YouTube videos and adherence to the adapted HONCode principles (Research Question 2). Binary logistic regression analyses were run to examine the influence of physical literacy YouTube video content properties on the quality of information (Research Question 3a) and viewer attitudes (Research Question 3b). Likewise, binary logistic regression analyses were run to determine the potential influence of the content delivery style of the physical literacy YouTube videos on the quality of information (Research Question 4a) and viewer attitudes (Research Question 4b).

### 2.3. Results

Demographic data and frequencies can be found in Table 2. However, prior to sharing the results of the research questions, we wanted to highlight several of the more critical aspects of the YouTube videos on physical literacy. The most prominent physical literacy properties covered in the YouTube videos were those centered on physical constructs (*n* = 122; 81.3%), followed closely by psychosocial and attitudinal concepts (*n* = 117; 78.0%), and behavioral aspects (*n* = 111; 74.0%). Affective components of physical literacy were present in the majority of the YouTube videos (*n* = 91; 60.7%), while the cognitive constructs were discussed in less than half of the videos (*n* = 67; 44.7%). Over half of the videos addressed these physical literacy domains in the context of unstructured play and physical activity (*n* = 84; 56.0%) with only 28 (18.7%) considering physical literacy in the context of structured sport and physical activity. The physical education space was the predominant context for 43.3% (*n* = 65) of the videos. Of the remaining 85 YouTube videos in which the information was presented outside the context of physical education, 38 (44.7%) were solely within the space of unstructured play, while one (1.2%) video focused only on structured sport. Thirty (35.3%) of these videos did not communicate information within the context of structured or unstructured play, while 16 (18.8%) videos considered both contexts.

The media sources uploading these physical literacy videos to YouTube were diverse. The two largest media sources of content comprised almost two-thirds of the videos: user-generated (*n* = 47; 31.3%) and non-profit organizations (*n* = 46; 30.7%). Nearly one-third of the YouTube videos were uploaded by a government agency/organization (*n* = 18; 12.0%), while educational institutions posted only eight videos (5.3%). Regarding the audiences of the YouTube videos, over half (*n* = 84; 56%) portrayed physical literacy as a lifelong journey and were inclusive of both youth and adults. Forty nine (32.7%) videos specifically addressed physical literacy in youth, while only 12 (8%) were directed solely towards an adult population. Only five (3.3%) of the YouTube videos were judged to not have an intended audience.

#### 2.3.1. Research Question 1: Is the Information Quality of YouTube Videos on Physical Literacy Associated with Viewer Attitudes?

Less than half of the YouTube videos were rated as being of high quality (*n* = 64; 42.7%), leaving the remaining 86 videos (57.3%) to be rated low quality. Principle #2, which requires videos to be clear in their mission to supplement and not replace information from certified/qualified educators and sources, was achieved by the most videos (*n* = 106; 70.7%). Principle #3, requiring videos to provide information and instruction in line with the most commonly accepted and understood conceptualizations of physical literacy, was the second most achieved (*n* = 91; 60.7%), while Principles #1 and #4, which required source information (*n* = 80; 53.3%) and credentials (*n* = 74; 49.3%), were each followed by nearly half of the videos. For further detail on the HONCode principles see Table A1. Results from the Mann-Whitney *U* test revealed a significant difference in the number of viewer likes given to low versus high quality YouTube videos, *U* = 3,377.50, *z* = 2.424, *p* = 0.015, *r* = 0.20, such that high quality videos, or those adhering to 4 or more adapted HONCode principles (*Md* = 3.00, *n* = 64), received more “likes” than low quality videos (*Md* = 1.00, *n* = 86).

#### 2.3.2. Research Question 2: Does the Quality of Physical Literacy Information in the YouTube Videos Vary Based on Media Source?

Due to 4 of the 14 cells (28.6%) containing less than the expected count of 5, Fisher’s exact test was conducted. The analysis revealed a statistically significant difference in adherence to the adapted HONCode principles based on the media source, Fisher’s exact test = 39.36, *p* < 0.001. The greatest number of high quality videos, per adapted HONCode principles, were posted by non-profit organizations with 26 (56.5%). While this was just above half of the videos posted by such social media accounts, the largest proportion of high-quality videos were posted by professional associations (*n* = 12; 85.7%;). The vast majority of user-generated YouTube videos on physical literacy were of low-quality (*n* = 41; 87.2%).

#### 2.3.3. Research Question 3a: Do the Content Properties in the YouTube Videos on Physical Literacy Influence the Quality of Information?

Binary logistic regression revealed that the physical literacy properties of the YouTube videos had a significant association with the quality of the videos, *x*^2^ (5, *N* = 150) = 49.73, *p* < 0.001. The full model explained between 28.2% (Cox & Snell *R* square) and 37.9% (Nagelkerke *R* square) of the variance in quality, per the HONcode principles, correctly classified 72.7% of the videos. Table A2 shows that videos focused on physical activity behaviors were the strongest predictor of high quality ratings (Wald = 4.75, *df* = 1, 95% CI: 1.17–18.29, *p* = 0.029), with an odds ratio (OR) of 4.62. Videos containing affective content were also found to be a significant predictor (OR = 3.6) of a video being rated as high quality (Wald = 4.90, *df* = 1, 95% CI: 1.16–11.18, *p* = 0.027).

#### 2.3.4. Research Question 3b: Do the Content Properties in the YouTube Videos on Physical Literacy Influence Viewer Attitudes?

Binary logistic regression revealed no significant findings regarding the influence of the physical literacy content properties and viewer attitudes.

#### 2.3.5. Research Question 4a: Does the Content Delivery Style of the Physical Literacy YouTube Videos Influence the Quality of Information?

Binary logistic regression revealed that the delivery style on the YouTube videos’ content had a significant impact on the quality of the videos, *x*^2^ (4, *N* = 150) = 42.56, *p* < 0.001. The full model explained between 24.7% (Cox & Snell *R* square) and 33.2% (Nagelkerke *R* square) of the variance in quality, per the HONcode principles, correctly classifying 73.3% of the videos. Table A2 shows that videos delivering content using a presentation style were the strongest predictor (OR = 13.7) of a video achieving high quality status (Wald = 27.30, *df* = 1, 95% CI: 5.13–36.5, *p* < 0.001). Delivering content via testimonial style was also a significant predictor of a high quality video (Wald = 10.01, *df* = 1, 95% CI: 1.77–11.22, *p* = 0.002), with an OR of 4.5.

#### 2.3.6. Research Question 4b: Does the Content Delivery Style of the Physical Literacy YouTube Videos Influence Viewer Attitudes?

The full model did not significantly explain any influence of the content delivery style of the physical literacy YouTube videos on viewer attitudes. However, videos delivering content through demonstration were found to be the strongest predictor, with an OR of 2.5, of viewers attitudes to “like” a video (Wald = 4.44, *df* = 1, 95% CI: 1.07–5.87, *p* = 0.035).

## 3. Discussion

The purpose of this study was to assess the content, exposure, engagement, and information quality of videos uploaded to YouTube, one of the most popular social media websites on the Internet [48]. More specifically, we sought to determine the qualities of videos as they relate to introducing, educating and serving the public as a shared resource(s) on physical literacy. What follows is a discussion of the results and implications of the data analysis.

### 3.1. Content and Delivery

When considering the value of physical literacy videos uploaded to YouTube, it is important to consider the content of said videos as well as the audience to whom the videos are directed. An overwhelming number of videos, 133, addressed physical literacy with consideration to youth while 96 targeted adults. This was an encouraging finding given that enhancing physical literacy should be a lifelong learning opportunity [63,64] that positively contributes to an enduring lifestyle of physical activity [56,65]. While physical literacy education and training seem inherently entangled in a pedagogical focus among physical educators [11,12], self-examination of one’s movements and interaction with the environment and developing into a physically literate individual can take place in a variety of spaces, as evidenced by the diverse contexts in which the videos were recorded. Therefore, it is important for health and activity educators and practitioners, as well as individuals, to consider, communicate, and embrace physical literacy in the context of fluctuating environments during and well beyond youth.

Over half of the videos demonstrated the concept of physical literacy through unstructured play or free play. Unstructured play and/or activities typically occur as time permits, during which participants determine the rules, (dis)organization, as well as goals and intentions of their activities, play, and movement without the restrictions of developmental plans that may inhibit their expression and enjoyment [66]. The value of free or unstructured play is critical in the development of athletic skills, movement, health and physical activity [67,68] and can positively impact one’s, particularly youth, progression toward becoming physically literate. Unfortunately, as evidenced by nearly one-third of the videos, physical literacy is often considered in the context of structured sport and physical activities or physical education sites. However, given that more videos placed physical literacy in the context of unstructured environments advocates for physical literacy as an individual lifelong journey. Thus, physical literacy instruction should reflect this journey by addressing physical pursuits and activities throughout the lifecourse, from early stages of physicality to adulthood [9]. As witnessed prior, the physical literacy videos posted on YouTube demonstrate this life course perspective of physical literacy, appropriately articulating and disseminating information to viewers as lifelong learners.

Given the breadth of one’s lifelong progression towards physical literacy, a number of topics were presented in the YouTube videos on physical literacy. Being a construct largely focused on movement and one’s ability to engage with the physical environment through a variety of movement forms, competencies and patterns [11,69], it was not surprising that most videos sought to educate viewers on elements of movement in the development of physical literacy. However, far too many videos failed to account for the wide array of physical skills and abilities that youth sport participants must masters. For physical literacy to advance towards becoming a universal health consideration for youth, one’s subjective understanding of physical capacity to move must be considered. It is of likewise importance for physical educators and organizations to consider the factors that may hinder youth from becoming physically active. Thus, it becomes critical that physical literacy is understood as an individualized journey across one’s life course as dictated by not only embodied interactions and potential, but also, institutional and structural constraints [8,70], many of which are not addressed when promoting physical literacy to youth.

The least focused on elements of physical literacy, although still present in over half of the videos, were those of affect and cognition. While not as salient as psychosocial and health-related behaviors, it is critical that video content focus more on the development of one’s confidence, motivation, and especially self-esteem, as a lack any or all of these constructs may hinder one’s disposition towards lifelong physical activity [8]. Likewise, when videos promote the continuing accumulation (or lack thereof) of physical literacy’s affective and behavioral components, they begin to cultivate a foundation from which youth, in particular, can value and take personal responsibility for the lifetime and long-term positive impacts on a healthy lifestyle [9]. However, this is not to underscore our prior points concerning the relative power of embodied potential and structural constraints to the subsequent maintenance of physical literacy. Rather, to cultivate this foundation whereby individuals, especially youth, are provided the agency to lead physically active lives and henceforth internalize the value of such a lifestyle, is to stay vigilant. Along these same lines, the positive social aspects of physical literacy were evidenced in the videos. Content reinforced the development and strengthening of social networks and support systems through interactions with others, particularly in the formative years of children and adolescents, which is of vital importance [10].

### 3.2. Quality

Intended as a code of ethics, the comprehensive set of principles put forth by the HONcode benefits both users and managers of online content regarding objective, verifiable, ethical, transparent and trustworthy medical and health-related information. However, not even half of the videos were deemed to be of high quality, as providers of information may not have shared their health and education qualifications for providing such information or share the source from which they collected their information. Yet, the intended mission of many videos was simply to educate viewers, and as such, it was made clear that physical literacy information was being communicated in accordance with commonly accepted definitions or understandings and as a supplement, not a replacement, to the advice of a qualified physical education or health source. Regardless, as a result of these findings, it is suggested that developers and uploaders of YouTube videos on physical literacy better source their information and be more transparent with their credentials via disclaimers espousing the adherence to the HONcode.

Regarding the quality of information, it was discovered that the greatest proportion (85%) of high-quality videos belonged to professional associations, despite the largest number of high quality videos being posted by non-profit organizations. Further, the lowest number of high-quality videos were of the user-generated variety, who conversely, were also responsible for the highest number and greatest proportion of low-quality videos. This suggests not only the value that viewers place on reputable sources of information, but also the preference and trust they may have towards reputable professional and non-profit organizations. Given the scarcity of high-quality and excess of low-quality videos posted by individual YouTube users, it was promising to see the potential apprehension viewers might have towards sources of information coming from individuals and non-credentialed groups or organizations. Thus, when users are being instructed or advised to use YouTube for additional information, it is recommended that they be directed to physical literacy videos from reputable sources that post high quality videos.

Surprisingly, educational institutions posted only eight (5.3%) videos. This is troubling given the knowledge and research capabilities of people and working groups within such spaces. Furthermore, as a legitimate health outcome and antecedent, physical literacy serves as a cross-disciplinary interest in a number of related areas of study. However, it is rebranded so as to better fit the purpose and understanding within each discipline [71], adding further confusion to understanding and application within and among multiple fields of study [72]. Additionally, there exists an extensive number of characteristics said to be associated with physical literacy [73], which may be too numerous for a single educational entity to assume definitive expertise. To this point, institutions in Canada have combined efforts of multiple institutions to arrive at a consensus definition and develop curriculum and programming accordingly [73]. Educational institutions may also feel hesitant to create and upload physical literacy videos to YouTube due to the lack of academic curriculum that can appropriately teach physical literacy, per educational standards and expectations, to students and knowledge seekers that range in age, abilities, interest and learning styles [74,75]. Yet, as knowledge purveyors, it is incumbent upon academicians within these types of programs to share their information to a more diverse population of races, genders, age, and socioeconomic status. These efforts will ensure a more inclusive, physically literate society. For instance, Pew Research data suggests that Black youth are not only more frequent users of Internet and social media platforms, but also more likely to use smartphones for Internet access as compared to White youth [76]. However, while social media usage provides some benefits to teenagers, such as opportunities for interpersonal and individualized learning, increased social awareness, and greater digital literacy, Black youth continue to be subject to lower levels of digital literacy and technological skill development [77]. Taken together, physical literacy educators and researchers should view this disparity between digital media use and digital fluency as an opportunity to engage with and educate diverse viewers of online content and youth.

As it concerns the availability of online teaching and learning opportunities, as well as the sharing of knowledge and resources, there are several findings that speak to best practices for content and delivery of physical literacy videos on YouTube. Regarding the quality of videos, providing content in either a formal or informal presentation has the greatest association with adherence to the HONcode principles. As such, health-related information disseminated in this format is judged to be the most trustworthy and in accord with, yet not disparately impacted by, common physical literacy knowledge and trends. Similarly, physical literacy videos with personal testimonies could reach viewers in a more open and honest way, such that they are more likely to be deemed high quality than other content delivery methods. However, YouTube viewers were more than twice as likely to “like” videos in which the information was delivered as a demonstration or instruction. This is understandable given the large focus (122 videos) on physical abilities and FMS in this study’s sample; as it is likely easier to understand and learn movement skills through visualizations including role models.

Lastly, videos that focused on the affective and behavioral components of physical literacy were judged as having the highest quality. Perhaps this has to do with the expertise warranted to speak appropriately and applicably on these components. This is not meant as a value judgment on the remaining core themes [10], but rather, these findings speak to the importance of understanding one’s confidence, motivation, and self-esteem in relation to their level(s) of and behavior towards physical activity. One’s mental health and physical well-being are not to be taken lightly, and speaking on the subject(s) requires a level of professionalism, knowledge and (possibly) credentials. The Internet is seemingly aware of this as uploaders of such content made sure they and their content were of high quality (e.g., ethical, trustworthy, transparent), affording them opportunities to speak to the affective and behavioral components that develop and facilitate a healthy and active lifestyle.

### 3.3. Study Limitations

Having to utilize YouTube’s integrated search engine, there is a chance that relevant videos on the website were not included in this study. Likewise, not being privy to the procedures by which engagement and evaluative metrics are determined and measured, we had to rely on YouTube’s controlled information in our assessment of physical literacy videos. Additionally, the information shared by uploaders of the videos was not always readily available or indicative of intentions leading to the creation and sharing of the physical literacy videos. Some of this limitation can be mitigated by collecting data on more videos and for a longer period of time. Other user metrics could also be collected to provide a more holistic view of user perceptions and quality of the uploaded videos. A longitudinal study in which viewer metrics are analyzed over a period of time could provide researchers and practitioners with better understandings of how and why videos trend, are sustained, and/or not viewed. Further, while this study was not limited to YouTube videos uploaded or viewed by users in a particular country, the use of the English language as inclusion criteria likely served to keep relevant, non-English speaking videos from being accounted for and coded.

From a design standpoint, several of the content and delivery coding procedures could serve to limit the interpretation of results. First, the coding of the content delivery style was not mutually exclusive. For instance, developers of a video could utilize both animation and presentation styles to send their message. Similarly, when considering the context of the videos (athletic or education, structured vs unstructured), both styles of play could be present in the videos, which could also be set in both environments. Thus, it is important to delve further into the role of structured vs unstructured play in both athletic and education contexts. Additionally, simply coding data collected from uploaded YouTube videos provides a limited perspective on site as an information resource for physical literacy. Future studies would benefit from taking a behavioral approach and collect self-reported data from watchers of the uploaded videos and users of YouTube to gain a stronger sense of their perceptions of the video website as an information resource.

Although well accepted by many organizations, the instrument is not without its shortcomings. As a self-regulatory tool that relies on codes of ethics, the HONcode may be limited in the sense that “self-regulation does not deter the unscrupulous, those who mostly need to have their ethical standards raised [78] (p. 236)”. Specifically, it has been noted that studies utilizing HONcode reported incorrect data as it concerned criteria adherence and assessment [79,80,81]. Nevertheless, we submit said limitations of the HONcode principles, for instance, to be less a reflection of the instrument as an unfit measure of ensuring high ethical standards and more so the result of either limited accountability or a lack thereof on the part of web sites engaged in distributing health-related content. While the purview of the present study is narrower in that it is centered on content distribution by way of video, we believe the merits of the HONcode principles, as opposed to similarly constructed instruments, to be in alignment with the purposes of this study.

## 4. Conclusions

As researchers and practitioners alike, we must continue to concern ourselves with the quality of health-related information available on the Internet, particularly YouTube, and other social media outlets. As it relates to online information resources and videos for physical literacy, it is critical to keep in mind who is watching these videos, how the information is being conveyed, in what context is it delivered, if is it influenced by a particular type of curricula (i.e., educational), and whether or not consideration was made towards certain socio-demographic factors and inclusivity. Lastly, what are the social determinants of physical literacy for youth? While the aforementioned list of considerations is by no means an exhaustive list, it challenges us to be mindful of the many intersecting factors that may not only be impacting one’s ability to become physically literate, but also the content, delivery, and quality of the resources intended to foster physical literacy in the first place. This study revealed that the content and content delivery method were most important in quality evaluations. Physical literacy videos that focused on physical activity and behaviors were the strongest predictor of high quality ratings, followed closely by videos covering affective domains (motivation, confidence, and self-esteem) of physical, literacy. The content delivery method was also important, with videos utilizing presentations and testimonials containing high quality information about physical literacy. Thus, providers of physical literacy and health-related online video content should be aware of and adhere to the expected quality standards.

The Internet is a resourceful educational space for health-related information and content [82,83]. Understanding physical literacy as a lifelong health-related outcome and facilitator of an active lifestyle, we sought to assess the content, exposure, engagement, and information quality of uploaded physical literacy video discussions, presentations, demonstrations and tutorials made available through YouTube. Our findings were encouraging in that they suggest the online physical literacy community has a desire for quality physical literacy information and content. Likewise, our findings speak to the quality standards expected of providers of health-related online video content. As expectations and ethical standards increase, the Internet, and specifically YouTube, has the potential to enhance video resources, virtual networking opportunities, as well as the sharing, dissemination, accumulation, and enrichment of physical literacy information for all.

## Figures and Tables

**Table 1:** Interrater reliability scores for coding content and other focus areas in sample (*n* = 40) of physical literacy videos on YouTube.

Content & Focus Areas	Cohen’s ĸ
**Properties of Physical Literacy**	0.82
*Affective*	0.86
Confidence	0.94
Motivation	0.74
Self-Esteem	0.80
*Cognitive*	0.62
Knowledge/Understanding	0.71
Value/Responsibility	0.46
*Physical*	0.82
Fundamental Movement Skills	0.73
Competence	0.85
*Behavioral*	0.86
Health	0.80
Physical Activity	0.92
*Psychosocial and Attitudinal*	0.85
Academic Performance	0.91
Enjoyment	0.82
Support	0.74
**Target Audiences**	0.90
Youth	0.84
Adults	0.84
None	N/A
**Contexts**	0.77
Physical Education	0.75
Structured Sport	0.80
Unstructured Sport	0.71
**Style of Content Delivery**	0.83
Animation	0.88
Demonstration	0.85
Presentation	0.86
Testimonial	0.65

**Table 2:** Frequencies of physical literacy content, topics, focus, and intent observed in sample of reviewed YouTube videos (*n* = 150).

Content & Focus Areas	*n*	Percentage
**Properties of Physical Literacy**		
*Affective*	91	60.7%
Confidence	87	58.0%
Motivation	68	45.3%
Self-Esteem	17	11.3%
*Cognitive*	67	44.7%
Knowledge/Understanding	64	42.7%
Value/Responsibility	37	24.7%
*Physical*	122	81.3%
Fundamental Movement Skills	112	74.7%
Competence	74	49.3%
*Behavioral*	111	74.0%
Health	57	38.0%
Physical Activity	109	72.7%
*Psychosocial & Attitudinal*	117	78.0%
Academic Performance	27	18.0%
Enjoyment	95	63.3%
Support	78	52.0%
**Target Audiences**		
Youth	133	88.7%
Adults	96	64.0%
None	5	3.3%
**Contexts**		
Physical Education	65	43.3%
Structured Sport	28	18.7%
Unstructured Sport	84	56.0%
**Style of Content Delivery**		
Animation	12	8.0%
Demonstration	74	49.3%
Presentation	56	37.3%
Testimonial	97	64.7%

The summation of content areas might add up to more than the number of videos in each properties because videos could contain more than one topic.

## Appendix A

**Table A1:** Adapted HONcode principles rating information quality in sample of reviewed YouTube videos (*n* = 150).

Number	Description of Criteria	Video Adherence	
		*n*	Percentage
1	The video provides physical education or health-related information and/or advice given by professionals or organizations whose qualifications/training are displayed. A disclaimer must be made whenever physical education or health-related information and/or advice is offered by a non-qualified source.	80	53.3%
2	The intended mission of the video is to provide information that supports the development and/or self-management of the physically literate individual. If/when applicable, a disclaimer must be made indicating that any information presented within the video is not meant to complement or replace the advice of a qualified physical education or health source.	106	70.7%
3	The notion of physical literacy must be found in accordance with commonly accepted definitions, as per qualified health and physical education professionals and organizations. For instance, SHAPE America accepts the following definition of physical literacy as “the ability to move with competence and confidence in a wide variety of physical activities in multiple environments that benefit the healthy development of the whole person.”	91	60.7%
4	For information presented in the video, a HTML link or bibliographic reference to external source data is provided. Additionally, the video makes readily accessible external source data, contact information, and/or an external link to a web site for further inquiry.	74	49.3%
5	Information provided in the video respects the privacy and confidentiality of all featured presenters, participants, and other individuals.	16	10.7%
6	For commercial or non-commercial organizations, as well as personal or private web sites, the source of either funds or materials presented in the video are clearly disclosed.	63	42.0%

**Table A2:** Associations between Video Quality, Likes, Style of Delivery, and Physical Literacy Properties (*N* = 150).

Style of Delivery & Content Properties	High Quality	Low Quality	OR	*p* Value	Liked	Not Liked	OR	*p* Value
	*n* (%)	*n* (%)	(95% CI)		*n* (%)	*n* (%)	(95% CI)	
Animation								
Yes	4 (2.7)	8 (5.3)	2.12	0.327	8 (5.3)	4 (2.7)	1.52	0.564
No	60 (40)	78 (52)	(0.47, 9.49)		96 (64)	42 (28)	(0.37, 6.32)	
Demonstration								
Yes	26 (17.3)	48 (32)	1.43	0.424	57 (38)	17 (11.3)	2.5	0.035 *
No	38 (25.3)	38 (25.3)	(0.60, 3.40)		47 (31.3)	29 (19.3)	(1.07, 5.87)	
Presentation								
Yes	40 (26.7)	16 (10.7)	13.68	0.000 ***	41 (27.3)	15 (10)	1.78	0.194
No	24 (16)	70 (46.7)	(5.13, 36.5)		63 (42)	31 (20.7)	(0.75, 4.26)	
Testimonial								
Yes	47 (31.3)	50 (33.3)	4.46	0.002 **	62 (41.3)	35 (23.3)	0.59	0.212
No	17 (11.3)	36 (24)	(1.77, 11.22)		42 (28)	11 (7.3)	(0.26, 1.35)	
Affective Properties								
Addressed	56 (37.3)	35 (23.3)	3.60	0.027 *	65 (43.3)	26 (17.3)	0.48	0.198
Not Addressed	8 (5.3)	51 (34)	(1.16, 11.18)		39 (26)	20 (13.3)	(0.16, 1.46)	
Cognitive Properties								
Addressed	43 (28.7)	24 (16)	1.42	0.465	50 (33.3)	17 (11.3)	1.45	0.454
Not Addressed	21 (14)	62 (41.3)	(0.55, 3.66)		54 (36)	29 (19.3)	(0.55, 3.80)	
Physical Properties								
Addressed	62 (41.3)	60 (40)	2.91	0.213	90 (60)	32 (21.3)	2.43	0.103
Not Addressed	2 (1.3)	26 (17.3)	(0.54, 15.65)		14 (9.3)	14 (9.3)	(0.84, 7.08)	
Behavioral Properties								
Addressed	61 (40.7)	50 (33.3)	4.62	0.029 *	82 (54.7)	29 (19.3)	1.66	0.328
Not Addressed	3 (2)	36 (24)	(1.17, 18.29)		22 (14.7)	17 (11.3)	(0.60, 4.56)	
Psychosocial & Attitudinal Properties								
Addressed	57 (38)	60 (40)	1.58	0.415	84 (56)	33 (22)	1.51	0.372
Not Addressed	7 (4.7)	26 (17.3)	(0.53, 4.75)		20 (13.3)	13 (8.7)	(0.61, 3.73)	

*Notes*: * *p* < 0.05, ** *p* < 0.01, *** *p* < 0.002.
